# From State-to-Trait Meditation: Reconfiguration of Central Executive and Default Mode Networks

**DOI:** 10.1523/ENEURO.0335-18.2019

**Published:** 2019-12-02

**Authors:** C. C. C. Bauer, S. Whitfield-Gabrieli, J. L. Díaz, E. H. Pasaye, F. A. Barrios

**Affiliations:** 1 McGovern Institute for Brain Research, MIT, Cambridge 02139, MA; 2 Instituto de Neurobiología, Universidad Nacional Autónoma de México, Querétaro, QRO 76230, México; 3 Cognitive Neuroscience Laboratory at Boston VA Healthcare System, Brockton 02130, MA; 4 Department of Psychology, Northeastern University, Boston 02115, MA; 5 Facultad de Medicina, Universidad Nacional Autónoma de México, Ciudad de México 04510, México

**Keywords:** experienced meditators, fALFF, functional connectivity, mind-wandering, mindfulness, top-down control

## Abstract

While brain default mode network (DMN) activation in human subjects has been associated with mind wandering, meditation practice has been found to suppress it and to increase psychological well-being. In addition to DMN activity reduction, experienced meditators (EMs) during meditation practice show an increased connectivity between the DMN and the central executive network (CEN). However, the gradual change between DMN and CEN configuration from pre-meditation, during meditation, and post-meditation is unknown. Here, we investigated the change in DMN and CEN configuration by means of brain activity and functional connectivity (FC) analyses in EMs across three back-to-back functional magnetic resonance imaging (fMRI) scans: pre-meditation baseline (trait), meditation (state), and post-meditation (state-to-trait). Pre-meditation baseline group comparison was also performed between EMs and healthy controls (HCs). Meditation trait was characterized by a significant reduction in activity and FC within DMN and increased anticorrelations between DMN and CEN. Conversely, meditation state and meditation state-to-trait periods showed increased activity and FC within the DMN and between DMN and CEN. However, the latter anticorrelations were only present in EMs with limited practice. The interactions between networks during these states by means of positive diametric activity (PDA) of the fractional amplitude of low-frequency fluctuations (fALFFs) defined as $\bar{CEN\;fALFF}\;-\;\bar{DMN\;fALFF}$ revealed no trait differences but significant increases during meditation state that persisted in meditation state-to-trait. The gradual reconfiguration in DMN and CEN suggest a neural mechanism by which the CEN negatively regulates the DMN and is probably responsible for the long-term trait changes seen in meditators and reported psychological well-being.

## Abstract (precis)

While brain default mode network (DMN) activation in human subjects has been associated with mind wandering, meditation practice has been found to suppress it and to increase psychological well-being. In addition to DMN activity reduction, experienced meditators (EMs) during meditation practice show an increased connectivity between the DMN and the central executive network (CEN).

## Significance Statement

We introduce a positive diametric activity (PDA) metric to measure the relation between two anticorrelated rs-fMRI networks in experienced meditators (EMs). PDA as well as functional connectivity (FC) are increased during meditation compared to resting state and persists in the post-meditation resting state. Conversely, meditation trait is characterized by positive PDA but with significant reduction in activity and FC within default mode network (DMN) and increased anticorrelations between DMN and central executive network (CEN). PDA and FC between DMN and CEN distinguish meditation state-to-trait effects.

## Introduction

What does the mind do when all physiologic needs are taken care of and there are no immediate demands? Does it part into an empty void, a dormant state to conserve resources until something disturbs it and activity is needed? We all know that this is not the case. Every moment of our waking experience challenges this explanation of mental life. When nothing requires active cogitative processing, our mind generally tends to think about what is not going on in the present moment, i.e., it wanders, recapitulates events that happened in the past, or fantasizes about what might happen in the future, jumping from one thought to the next with amazing speed and apparent randomness (Mason et al., 2007). Undeniably, this never stopping stream of consciousness appears to be our brain’s idle mode of being, our default mode when not otherwise engaged (Mason et al., 2007; Christoff et al., 2016; Kane et al., 2017). Although this ability is as an evolutionary achievement that allows us to learn, reason, and plan (Mantini et al., 2013; Rilling, 2014), it swiftly fails to serve its adaptive function, and turns into a risk factor for health and psychological well-being whenever it becomes a rigid and inflexible pattern with tremendous emotional costs (Smallwood et al., 2009; Killingsworth and Gilbert, 2010; Ottaviani et al., 2013; Simon and Engström, 2015; Jazaieri et al., 2016). Consequently, the underlying mental processes that keep the brain active when not otherwise engaged have been a source of significant theoretical surmise. Since the advent of neurophysiological recordings, it has been determined that the brain is never truly at rest (Berger and Hans, 1933). From a functional imaging perspective, the remarkable properties of the brain’s intrinsic activity were first noted by Biswal et al. (1995). They observed that the “noise” in the spontaneous functional magnetic resonance imaging (fMRI) blood oxygen level-dependent (BOLD) signal exhibited striking patterns of spatial coherence corresponding, in their case, to the sensorimotor regions of the cerebral cortex. Subsequently, Raichle and colleagues first took notice that during the commonly used “baseline” in research paradigms, the synchronous behavior of a number of anatomic regions were observed to deactivate during task and thus initially identified as a network of task-specific deactivations, later named the default mode network (DMN; Gusnard et al., 2001; Buckner et al., 2008). Soon it was determined that these task specific deactivations of the DMN also showed patterns of coherent activation during periods of rest that included anterior (medial prefrontal cortex, MPFC) and posterior midline structures (posterior cingulate, PCC) as well as lateral temporal cortex (LTC) and the hippocampus. Additionally, in healthy individuals, DMN activity has been shown to be anticorrelated (negatively correlated) with brain regions activated during attention demanding tasks (e.g., the central executive network, CEN; Greicius et al., 2003; Fox et al., 2005; Fransson, 2005; Kelly et al., 2008; Uddin et al., 2009). Specifically, the CEN, typically including the dorsolateral prefrontal cortex (DLPFC) and posterior parietal cortex (PPC), supports these attention demanding tasks, i.e., attentional control and working memory. Across individuals greater magnitude of DMN-CEN anticorrelations is associated with superior cognitive function such as complex working memory (Whitfield-Gabrieli et al., 2009; Hampson et al., 2010; Keller et al., 2015). Abnormal DMN activity, such as competitive, antagonistic DMN activation during CEN activity or changes in connectivity between subregions of the DMN, has also been associated not only with lower levels of happiness (Killingsworth and Gilbert, 2010; Smallwood and O’Connor, 2011), but with a number of psychological disorders such as anxiety (Zhao et al., 2007), depression (Sheline et al., 2009), schizophrenia (Garrity, 2007; Pomarol-Clotet et al., 2008; Whitfield-Gabrieli et al., 2009; Camchong et al., 2011; Bastos-Leite et al., 2015), epilepsy (Liao et al., 2011), autism (Assaf et al., 2010), attention deficit hyperactivity disorder (ADHD; Uddin et al., 2008), and Alzheimer’s disease (AD; Greicius et al., 2004; Sheline and Raichle, 2013). Recent studies further suggest that abnormal DMN activity and connectivity plays a role in neuropsychiatric disorders (Whitfield-Gabrieli and Ford, 2012; Raichle, 2015). These associations have led to the suggestion of using the DMN as method by which to study mental disorders, resulting in a growing body of literature concerning disorder-specific variations within the DMN (Arens et al., 2003; Greicius, 2008; Broyd et al., 2009; Fox and Greicius, 2010; Whitfield-Gabrieli and Ford, 2012; Simon and Engström, 2015).

Given the interrelationship between mind-wandering, DMN activity, and the risk to health and psychological well-being, a question arises: Is it possible to change this maladaptive mode into one that is more pleasant and healthier? According to many philosophical and contemplative traditions, yes, this is possible. Thus, these teach that happiness is to be found by “living in the moment,” i.e., to be here and now without losing oneself in past or future thought. For more than two millennia, meditation has been practiced as a means of achieving this ephemeral mind state, psychological equanimity, and self-awareness, yet it has only recently become the target of systematic Western-world research for its relevance to mental and physical health in fields such as medicine, psychology, and neuroscience (Van Dam et al., 2018). Undeniably, meditation is becoming increasingly well regarded for its therapeutic promise (Buchholz, 2015; Creswell, 2015; Gu et al., 2015; Simon and Engström, 2015) and meditation methods have been beneficial in the treatment of psychological disorders such as schizophrenia (Chien and Thompson, 2014), depression (Teasdale et al., 2000; Ma and Teasdale, 2004; Eisendrath et al., 2008; Kuyken et al., 2008; Yang et al., 2016), anxiety (Baer, 2003; Grossman et al., 2004; Ludwig and Kabat-Zinn, 2008; Shen et al., 2014), addiction (Bowen et al., 2014), alcoholism (Witkiewitz et al., 2005; Garland et al., 2010), smoking (Tang et al., 2013), mild cognitive impairment (MCI; Wells et al., 2013), and ADHD (Zylowska et al., 2008; Bueno et al., 2015; Janssen et al., 2015). Preliminary findings have suggested that the effects of meditation include better emotion regulation (Lutz et al., 2014; Turner, 2014; Prakash et al., 2015), self-regulation (Tang et al., 2014), awareness and self-perception (Hölzel et al., 2011b), memory and cognition (Zeidan et al., 2010), attention (Moore et al., 2012), working memory (Mrazek et al., 2013; Banks et al., 2015), as well as gray and white matter differences in experienced meditators (EMs; Luders et al., 2009; Hölzel et al., 2011a; Fox et al., 2014). Functional imaging studies on meditation practice have examined two distinct effects of meditation. The first, called a state effect, refers to the short-term consequences of meditation practice on the individual’s state. This would include short lived changes in bodily awareness, relaxation, emotion regulation, attention and in BOLD activation or functional connectivity (FC) when measured with fMRI. During this active state of meditation, studies have consistently found that within-network connectivity of the DMN as well as between-network connectivity of DMN, CEN and salience network (SAL) are increased (Brewer et al., 2011; Jang et al., 2011; Garrison et al., 2014; Jao et al., 2016). The second, called a trait effect, refers to long-lasting changes in these same dimensions, which continue after practice and during the meditator’s daily life (Lutz et al., 2007). However, the findings for this trait effect have not been so clear when measured with resting-state FC (rsFC), with mixed results (for review, see Mooneyham et al., 2016). Furthermore, little to nothing is known about the activity and connectivity change of DMN and CEN that intertwine the transitory state effects of meditation with the lasting trait effects of meditation practice.

Here, we hypothesized that activity of the CEN is an important modulator of DMN activity and connectivity, both during the meditation state and during the transition phase post-meditation. Support for this idea comes on the one hand from correlational neuroimaging studies looking into attentional and cognitive control (McKiernan et al., 2003; Fox et al., 2005; Fransson, 2005; Dosenbach et al., 2007; Northoff et al., 2007; Sonuga-Barke and Castellanos, 2007; Sridharan et al., 2008; Anticevic et al., 2012), but specifically from a study by Chen et al. (2013) that provides direct evidence for this neural mechanism. In their study, they specifically tested this mechanism by exciting or inhibiting nodes within the CEN using noninvasive brain stimulation and observed the results using simultaneous brain imaging. They found that the DMN is under inhibitory control specifically from a node in CEN (posterior middle frontal gyrus pMFG; BA 9/10). Accordingly, we predicted that brain activation in nodes of the CEN during meditation would: (1) modulate activity of the DMN and increase connectivity between DMN and CEN, (2) these changes would persist after meditation and finally, (3) that there are trait differences in DMN and CEN activity and connectivity characteristic of meditation experience. To test these predictions, we conducted three back-to-back fMRI runs in EMs: pre-meditation baseline (trait), meditation (state), and post-meditation (state-to-trait). The pre-meditation baseline was also performed on a group of healthy controls (HCs) as a comparison.

## Materials and Methods

### Participants

After standard exclusion criteria for fMRI research were applied, 16 EMs (six females, mean age 41.12 years, SD 10.5, average of 1677 ± 367 h of Vipassana meditation experience) and 17 meditation naive HCs (seven females, mean age 35.70 years, SD 4.7) participated in the study. There was no significant age difference between groups (*t*_(21)_ = –1.68, *p* = 0.10). All subject groups were recruited and scanned in the same time period as part of the same experiment and all subjects gave informed consent for the experimental procedure, and the protocol had IRB approval.

### Experiment design

BOLD fMRI data were collected from all participants during a 5-min, eyes-open resting-state period (rsBase). In addition, immediately following the rsBase scan, EM participants were also scanned during a 20-min, eyes-open period while engaged in Vipassana meditation (Med). Vipassana meditation emphasizes focused attention typically involving the deliberate focus of attention to a chosen target, such as general body sensations or sensations related to breathing, and the voluntary redirection of attention each time it lapses (Hart, 2011). Finally, EM underwent a second 5-min eyes-open resting-state period (rsPost).

### Meditation trait, state, and state-to-trait operationalizations (Austin, 1999; Shapiro and Walsh, 1984; West, 2016)

#### Meditation trait

**Figure 1.**
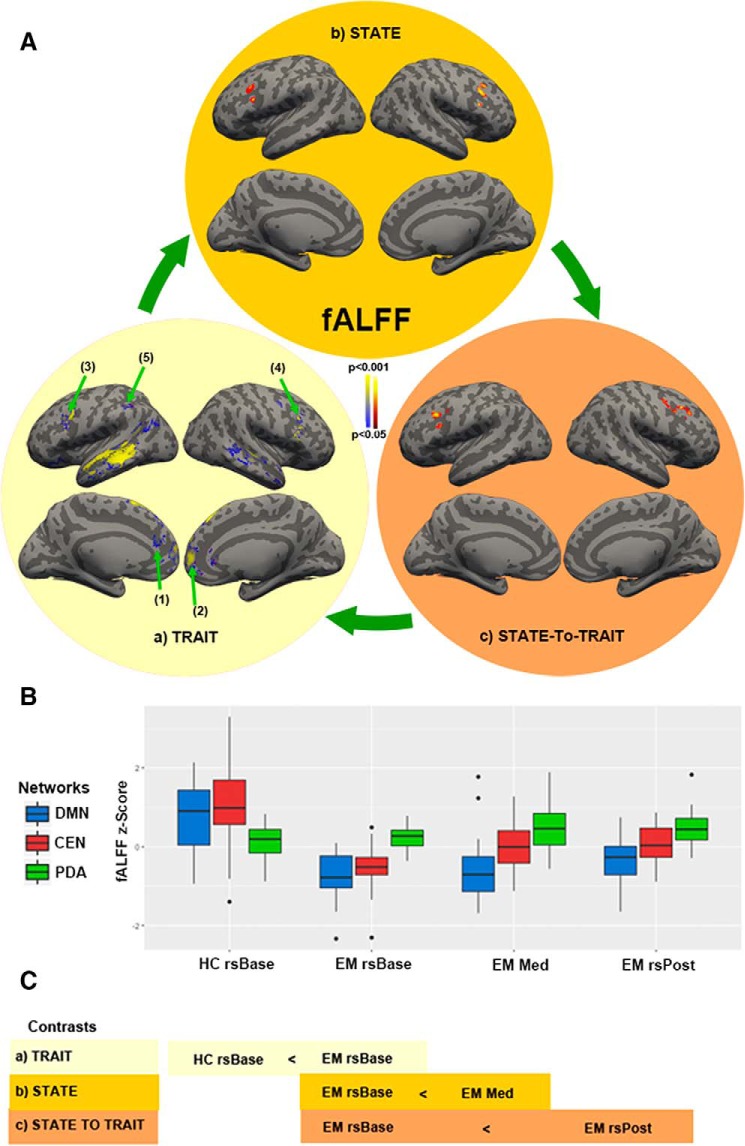
State-to-trait meditation brain activity changes of the fractional Amplitude of Low-Frequency Fluctuations (fALFFs). ***Aa***, Brain regions showing trait changes in fALFF between meditators and HCs at baseline (HC rsBase < EM rsBase). ***Ab***, Brain regions that show significant changes in fALFF during the meditation state in meditators (EM rsBase < EM Med). ***Ac***, Brain regions that show significant changes in fALFF during the transition from state-to-trait meditation in meditators (EM rsBase < EM rsPost). ***B***, Boxplot showing the mean fALFF *z*-scores in blue (DMN) and red (CEN) and PDA scores in green during baseline (rsBase), meditation (Med) and post-meditation (rsPost) for HCs and EMs. ***C***, Schematic representation of state-to-trait contrasts. All stats shown are nonparametric (5000 permutations) with height threshold *p* < 0.05 and cluster-size FDR-corrected *p* < 0.05. Black dots represent subjects that lie beyond the whiskers.

The lasting changes in sensory, cognitive, and self-referential awareness and their underlying brain activity and connectivity that persist in the meditator irrespective of being actively engaged in meditation. Contrast HC rsBase < EM rsBase (Fig. 1*Ca*).

#### Meditation state

Refers to the altered sensory, cognitive, and self-referential awareness that can arise during meditation practice and their underlying brain activity and connectivity. In the present study this will correspond to the contrast EM rsBase < EM Med (Fig. 1*Cb*).

#### Meditation state-to-trait

Refers to the changes in sensory, cognitive, and self-referential awareness and their underlying brain activity and connectivity that persist in the meditator after an active engaged meditation session. Contrast EM rsBase < EM rsPost (Fig. 1*Cc*).

### MRI data acquisition

MRI imaging was performed on a 3.0T GE MR750 instrument (General Electric) using a 32-channel head coil. Functional imaging for resting state included 35 slices, acquired using a T2*-weighted EPI sequence with TR/TE 2000/40 ms, a 64 × 64 matrix and 4-mm slice thickness, resulting in a 4 × 4 × 4 mm^3^ isometric voxel and a total of 151 volumes. For meditation imaging included 35 slices, acquired using a T2*-weighted EPI sequence with TR/TE 1500/40 ms, a 64 × 64 matrix and 4-mm slice thickness, resulting in a 4 × 4 × 4 mm^3^ isometric voxel and a total of 804 volumes. The shorter TR during meditation was chosen according to the parameters in Hasenkamp et al. (2012) to adhere to additional experiential sampling during meditation and thus needed a faster acquisition. Importantly, this discrepancy has been shown not to affect fractional amplitude of low-frequency fluctuation (fALFF) nor FC correlation coefficients (CCs) since multiple repetition times have been empirically compared to address the issue of temporal mismatch (Wu et al., 2011). Additionally, high-resolution structural 3D-T1-weighted images were acquired for anatomic localization (resolution of 1 × 1 × 1 mm^3^, TR = 2.3 s, TE = 3 ms) covering the whole brain. The images were acquired with an acceleration factor = 2.

### Preprocessing

The preprocessing of resting-state images was done using SPM 12 software (http://www.fil.ion.ucl.ac.uk/spm) implemented in a MATLAB suite (MathWorks, Inc.). It included slice time correction, head motion correction, co-registration to subjects’ structural images, segmentation, normalization, linear detrending, and smoothing (FWHM = 8 mm).

### Brain activity analysis

To detect regional brain activity changes we used an improved approach of the ALFFs method, fALFF (Zou et al., 2008 ). This method takes the ratio of power spectrum of low-frequency (here: 0.008–0.09 Hz) to that of the entire frequency range. fALFF analysis was conducted with AFNI’s 3dRSFC (Taylor and Saad, 2013 ). Similar to the procedures of previous literature (Zou et al., 2008; Shpaner et al., 2014; Kong et al., 2015), the time series of each voxel was transformed to a frequency domain after the linear trend was removed without bandpass filtering. The square root was then calculated at each frequency of the power spectrum, and finally the sum of amplitude across 0.008–0.09 Hz was divided by that across the entire frequency range (0–0.25 Hz for TR = 2 s and 0–0.33 Hz for TR = 1.5 s, see fMRI data acquisition) to obtain fALFF. Importantly, the TR discrepancy has been shown not to affect fALFF since multiple repetition times have been empirically compared to address the issue of temporal mismatch ( Wu et al., 2011 ). Next, we obtained the meditation trait differences of the fALFF maps of HC and EM at baseline (rsBase) using two-sample *t* tests. The thresholded images were then converted into binarized masks and were used as the target regions of interest (ROIs) from which to extract the mean fALFF maps for all subjects and states. All imaging analysis were performed with FSL’s randomize tool for nonparametric permutation (5000 permutations) inference on neuroimaging data (Winkler et al., 2014 ) and masked with the binarized DMN and CEN templates of Yeo et al. (2011 ) and threshold-free cluster enhancement (TFCE; Smith and Nichols, 2009 ) cluster corrected (*p* < 0.05) . We first obtained the meditation trait differences of the fALFF maps of HC and EM at baseline (rsBase) using two-sample *t* test and then extracted the mean fALFF maps for all subjects and states. The extracted values were converted to normally distributed *z*-scores to allow for second-level analyses using multilevel modeling one-way repeated measures ANOVA and *post hoc* Tukey’s test. Statistical analysis was performed with the open-source R package (www.R-project.org).

### Positive diametric activity (PDA)

To further assess the information processing during meditation, we introduce a PDA metric to determine the activation change of CEN and DMN according to their mean fALFF. The PDA metric is based on the hypothesis that there is a causal neural mechanism by which the CEN negatively regulates the DMN (Chen et al., 2013). This is to say that when CEN activity is increased this produces an inhibitory effect on the DMN and its activity is decreased. Accordingly, we define the PDA as follows:

$$PDA=\underline{CENfALFF}-\underline{DMNfALFF},$$

where PDA will always be positive as long as the mean activity of CEN is greater than the mean activity of DMN, reflecting precisely this negative relation between CEN and DMN. Meditation trait differences of the PDA for HC and EM at baseline (rsBase) was assessed using two-sample *t* tests. The PDAs for EM rsBase, Med, and rsPost were analyzed using multilevel modeling one-way repeated measures ANOVA and *post hoc* Tukey’s test.

### FC analysis

FC analysis was performed using a seed-driven approach with in-house, custom software CONN 16.b (Chai et al., 2012; Whitfield-Gabrieli et al., 2012). We performed seed-voxel correlations by estimating maps showing temporal correlations between the BOLD signal from the target ROIs and that of every brain voxel (i.e., whole-brain analysis). The specific target ROI clusters were obtained from the previous fALFF analysis two-sample *t* tests between HC and EM at rsBase for the DMN and CEN respectively (see above, Brain activity analysis). This yielded seed ROIs for the DMN in the medial prefrontal cortices (see clusters 1 and 2 in Fig. 1*Aa* and Table 1), which are nodes that have been implicated in processing of self-referential stimuli and in generating a model of the self (Northoff and Bermpohl, 2004) and for the CEN in bilateral inferior frontal gyrus (IFG) and inferior parietal lobule (IPL; see clusters 3, 4, and 5 in Fig. 1*Aa* and Table 1). Physiologic and other spurious sources of noise were estimated and regressed out using the anatomic CompCor method (aCompCor; Chai et al., 2012). Global signal regression, a widely used preprocessing method, was not used because it artificially creates negative correlations that prevent the interpretation of anticorrelation (Behzadi et al., 2007; Chai et al., 2012; Whitfield-Gabrieli et al., 2012) and can contribute to group differences in positive correlations (Saad et al., 2012). Instead, aCompCor allows for interpretation of anticorrelations and yields higher specificity and sensitivity compared with global signal regression (Chai et al., 2012). A temporal bandpass filter of 0.008 to 0.09 Hz was applied simultaneously to all regressors in the model. We used methods that minimize the influence of motion and artifact and that allow for valid identification of correlated and anticorrelated networks (Behzadi et al., 2007; Chai et al., 2012; Whitfield-Gabrieli et al., 2012). To address the spurious correlations in resting-state networks caused by head motion we used quality assurance software artifact detection tools (http://www.nitrc.org/projects/artifact_detect; http://www.nitrc.org/projects/conn; Whitfield-Gabrieli et al., 2012) to identify problematic time points during the scan. Specifically, an image was defined as an outlier if the head displacement in *x*, *y*, or *z* direction was greater than 0.5 mm from the previous frame, or if the global mean intensity in the image was >3 SDs from the mean image intensity for the entire resting scan. A single regressor for each outlier image was included in the first level general linear model along with motion parameters and first order derivatives (there were no significant differences between groups and runs; Fig. 1). The anatomic image for each participant was segmented into white matter, gray matter, and CSF masks using SPM 12. To minimize partial voluming with gray matter, the white matter and CSF masks were eroded by one voxel, which resulted in substantially smaller masks than the original segmentations (Chai et al., 2012). The eroded white matter and CSF masks were then used as noise ROIs. Signals from the white matter and CSF noise ROIs were extracted from the unsmoothed functional volumes to avoid additional risk of contaminating white matter and CSF signals with gray matter signals. Previous results showed that aCompCor signals were considerably different from the global signal, as regressing higher order principal components of the global signal diminished both positive and negative correlations whereas regressing aCompCor signals resulted in stronger anticorrelations and eliminated spurious correlations (Behzadi et al., 2007). Time series of all the voxels within each seed were averaged, and first-level correlation maps were produced by extracting the residual BOLD time course from each seed and computing Pearson correlation coefficients (CCs) between that time course and the time course of all other voxels. CCs were converted to normally distributed *z*-scores using the Fisher transformation to allow for second-level general linear model analyses. Meditation Trait differences of HC and EM at baseline (rsBase) were compared using two-sample *t* tests. Second-level analyses for EM rsBase, Med, and rsPost, were compared using with a one-way repeated measures ANOVA implemented in CONN. All contrasts are nonparametric (1000 permutations) with height threshold *p* < 0.05 and cluster-size FDR-corrected *p* < 0.05, unless otherwise stated.

**Table 1.** Differences in fractional Amplitude of Low-Frequency Fluctuations (fALFF) for the different meditation states

Region	BA	Voxels	MNI (*x*,*y*,*z* mm)	*p* value*
**Meditation trait effects**				
HC rsBase > EM rsBase				
Default Mode Network (DMN)				
Medial Temporal Gyrus	L 21	1302	–66,–34,–6	0.02
Medial Prefrontal Cortex	R 10	18	6,60,26	0.01
Medial Prefrontal Cortex	L 10	147	–2,58,12	0.05
Central Executive Network (CEN)				
Inferior Frontal Gyrus	L 9	405	–34,6,34	0.01
Inferior Frontal Gyrus	R 9	144	54,20,20	0.03
Inferior Parietal Lobule	L 40	74	–54,–38,50	0.05
**Meditation state effects**				
EM Med > EM rsBase				
Default Mode Network (DMN)				
No significant differences	N/A	N/A	N/A	N/A
Central Executive Network (CEN)				
Inferior Frontal Gyrus	R 9	115	46,18,30	0.02
Inferior Frontal Gyrus	L 9	82	–44,10,26	0.03
**Meditation state-to-trait effects**				
EM rsPost > EM rsBase				
Default Mode Network (DMN)				
No significant differences	N/A	N/A	N/A	N/A
Central Executive Network (CEN)				
Inferior Frontal Gyrus	L 9	17	–46,18,34	0.02
Inferior Frontal Gyrus	R 9	4	50,28,38	0.05

Brodmann areas (BA), number of voxels and Montreal Neurological Institute coordinates (MNI). *All statistics are nonparametric (5000 permutations) with height threshold *p* < 0.05 and cluster-size FDR-corrected *p* < 0.05.

### Correlation between PDA and FC

To assess whether there is a relationship between brain activity (fALFF) and FC, we correlated the individual PDA scores (see above) with differences in FC of DMN and CEN ROIs for the different meditation states.

## Results

### Meditation trait effects

#### fALFF analysis

##### DMN

EM showed reduced activity in the left medial temporal gyrus (MTG; BA 21), right superior frontal gyrus (SFG; BA 6), left MPFC; BA 10, Fig. 1*Aa1*,*Aa2*, blue box plots in Fig. 1*B* (HC rsBase < EM rsBase) rs Base and Table 1.

##### CEN

EM showed reduced activity in bilateral DLPFC; BA 9, Fig. 1*Aa3*,*Aa4*; red box plots (HC rsBase < EM rsBase) in Fig. 1*B*; Table 1 and left IPL (BA 40, Fig. 1*Aa5*; Table 1). For this and all other fALFF significant MNI coordinates, see Table 1.

#### FC analysis

##### DMN

**Figure 2.**
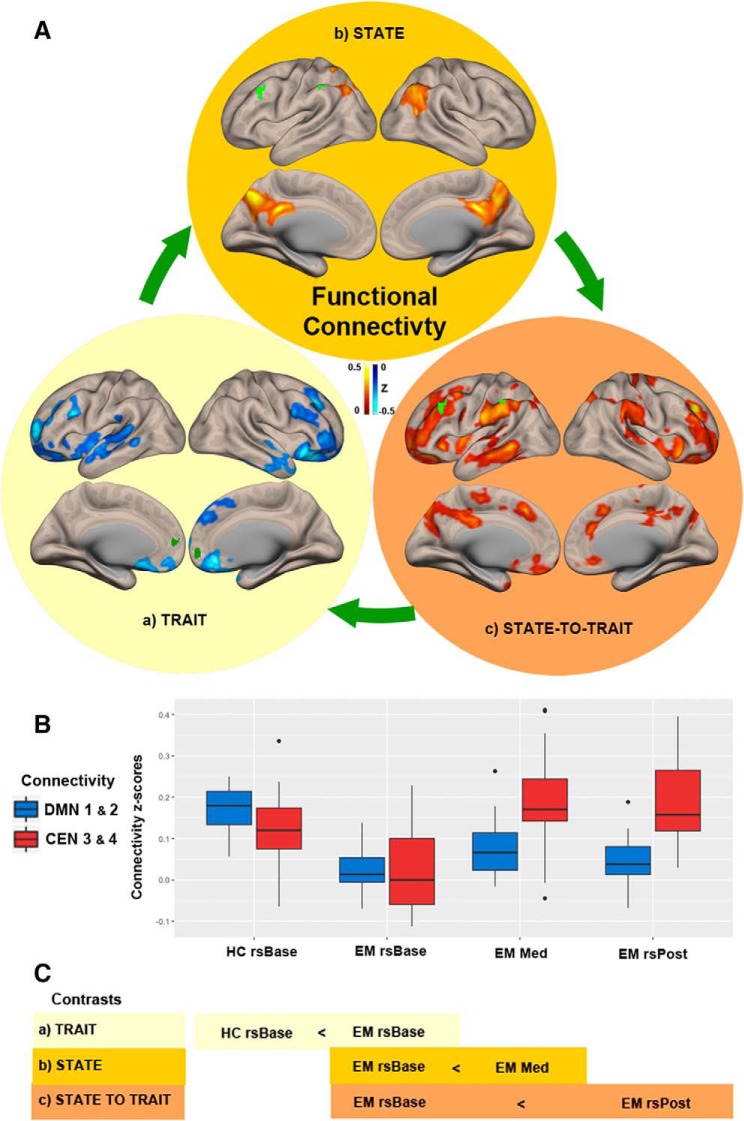
State-to-trait meditation functional connectivity (FC) changes. ***Aa***, Brain regions showing trait FC changes between experienced meditators (EM) and healthy controls (HCs) at baseline (HC rsBase < EM rsBase). ***Ab***, Brain regions that show significant FC changes during the meditation state in meditators (EM rsBase < EM Med). ***Ac***, Brain regions that show significant FC changes during the transition from state-to-trait meditation in meditators (EM rsBase < EM rsPost). Dark green (DMN ROIs 1 and 2) and light green (CEN ROIs 3 and 4) clusters show in each case the seeds used to determine the shown contrast (Fig. 1*Aa*). ***B***, Boxplot showing mean FC *z*-scores in blue (DMNs 1 and 2) and red (CENs 3 and 4) during baseline (rsBase), meditation (Med), and post-meditation (rsPost) for HCs and EMs. ***C***, Schematic representation of state-to-trait contrasts. All stats shown are nonparametric (1000 permutations) with height threshold *p* < 0.05 and cluster-size FDR-corrected *p* < 0.05. Black dots represent subjects that lie beyond the whiskers.

EM showed reduced connectivity with left SFG (BA 11), right medial frontal gyrus (MFG; BA 10), IPL (BA 40), and superior temporal gyrus (STG; BA 38), Fig. 2*Aa*; blue box plots (HC rsBase < EM rsBase) in Fig. 2*B* and Table 2. For this and all other significant connectivity coordinates, see Table 2.

**Table 2.** Differences in functional connectivity (FC) for the different meditation states

Region	Connectivity	BA	Voxels	MNI (*x*,*y*,*z* mm)	*p* value*
**Meditation trait effects**					
EM rsBase > HC rsBase					
Default Mode Network (DMN)					
Superior Frontal Gyrus	Reduced	L 11	>1000	–16,60,26	0.01
Middle Frontal Gyrus	Reduced	L 9	>200	–26,26,32	0.01
Inferior Parietal Lobule	Reduced	L 40	>200	–38,–48,26	0.02
Superior Temporal Gyrus	Reduced	R 38	>200	48,16,–20	0.03
Central Executive Network (CEN)					
No significant differences	N/A	N/A	N/A	N/A	N/A
**Meditation state effects**					
EM Med > EM rsBase					
Default Mode Network (DMN)					
No significant differences	N/A	N/A	N/A	N/A	N/A
Central Executive Network (CEN)					
Middle frontal gyrus	Increased	L 10	>200	–29,48,15	0.02
Middle frontal gyrus	Increased	R 10	>200	31,52,12	0.02
Anterior cingulate cortex	Increased	L 32	>200	4,30,24	0.02
Posterior cingulate cortex	Increased	R 31	>200	3,–31,38	0.03
Inferior Parietal Lobule	Increased	L 40	>200	–38,–46,46	0.03
**State-to-trait effects**					
EM rsPost > EM rsBase					
Default Mode Network (DMN)					
No significant differences	N/A	N/A	N/A	N/A	N/A
Central Executive Network (CEN)					
Precuneus	Increased	L 23	>1000	–2,–42,28	0.02
Angular gyrus	Increased	R 39	>1000	50,–66,33	0.02

Brodmann areas (BA), number of voxels and Montreal Neurological Coordinates (MIN). *nonparametric (1000 permutations) with height threshold *p* < 0.05 and cluster-size FDR-corrected *p* < 0.05.

##### CEN

No differences were found.

#### PDA analysis

No significant differences were found.

#### Correlations between PDA and FC

##### DMN

No significant correlations were found.

##### CEN

No significant correlations were found.

#### Correlations between hours of meditation practice and FC

##### DMN

No significant correlations were found.

##### CEN

Whole-brain FC analysis revealed a significant correlation (*R* = 0.87) between the hours of meditation practice and connectivity between CEN and MPFC at rsBase for EM (Fig. 4*A*).

To better understand this positive correlation between meditation experience and increased connectivity between CEN and MPFC, which also has been reported in other studies comparing novice versus expert meditators (Brewer et al., 2011; Jang et al., 2011; Shaurya Prakash et al., 2013), we divided our EM group into two sub-groups (median = 1130 h): EMs with >1130 h of daily practice (EM > 1130; approximately three years) and intermediate meditators (EM < 1130) and conducted a multilevel modeling one-way repeated measures ANOVA and *post hoc* Tukey’s test for the connectivity *z*-scores between CEN and MPFC. The results confirmed a significant difference between HC (mean = 0.13) and EM < 1130 (mean = –0.04, b = –0.16, *p* < 1e-3) but no difference between HC and EM > 1130 (mean = 0.04, b = –0.09, *p* = 0.08). Furthermore, there was also a significant difference between EM < 1130 (median = –0.25) and EM > 1130 (median = –0.07, b = 0.17, *p* = 0.04; Fig. 2).

#### Correlations between hours of meditation practice and PDA

No significant correlations were found for meditation trait, state, or state-to-trait PDA and meditation hours.

### Meditation state effects

#### fALFF analysis

##### DMN

No significant differences were found.

##### CEN

The multilevel modeling one-way repeated measures ANOVA yielded significant variation among rsBase, Med, and rsPost, χ^2^(2) = 24.57, *p* < 1e-04. The *post hoc* analysis showed that CEN fALFF was significantly increased in bilateral DLPFC (BA 9, Fig. 1*Ab*) during Med (mean = 0.3) compared to rsBase (mean = –0.55, b = 0.84, *p* < 1e-04). See red box plots (EM rsBase < Med) in Figure 1*B*.

#### FC analysis

##### DMN

No significant differences were found.

##### CEN

EM showed increased connectivity of posterior cingulate cortex (PCC; BA 31) and IPL; BA 40; Fig. 2*Ab* and red box plot (EM rsBase < Med) of Fig. 2*B*.

#### PDA analysis

The multilevel modeling one-way repeated measures ANOVA yielded that there was significant variation among rsBase, Med, and rsPost, χ^2^(2) = 12.54, *p* = 0.005. The *post hoc* analysis showed that PDA was significantly increased during Med (mean = 0.65) compared to rsBase (mean = 0.23, b = 0.57, *p* < 0.003) and rsPost (mean = 0.36, b = 0.42, *p* < 0.02). See green box plots (EM rsBase < Med) in Figure 1*B*.

#### Correlations between PDA and FC

##### DMN

No significant correlations were found.

##### CEN

**Figure 3.**
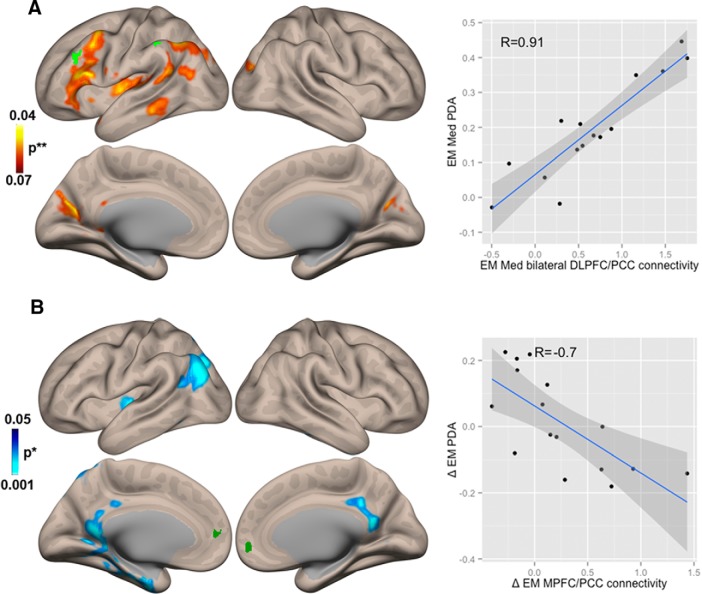
Correlations between Positive Diametric Activity (PDA) and Functional Connectivity (FC). ***A***, Brain regions that show significant correlation between PDA and FC during the meditation state in experienced meditators (EM) (EM Med). ***B***, Brain regions that show significant correlation between the change in PDA and change in FC during the transition from state-to-trait meditation (**Δ**EM = rsBase - rsPost). Dark green clusters at the Default Mode Network (DMN ROIs 1 and 2 from Fig. 1*Aa*) and bright green clusters at the Central Executive Network (CEN ROIs 3 and 4 from Fig. 1*Aa*) show in each case the seeds used to determine the estimated contrast. *nonparametric (1000 permutations) with height threshold *p* < 0.05 and cluster-size FDR-corrected *p* < 0.05; **parametric stats with height threshold *p* < 0.05 and cluster-size FDR-corrected *p* < 0.05.

There was positive correlation (*R* = 0.91, parametric stats with height threshold *p* < 0.05 and cluster-size FDR-corrected *p* < 0.05) between PDA during Med and FC during Med of bilateral DLPFC (BA 9) and left posterior cingulate cortex (PCC; BA 31), left IFG (BA 45), left STG (BA 22), and left inferior parietal lobe (IPL; BA 40; Fig. 3*A*). For this and all other significant correlation analysis coordinates, see Table 3.

**Table 3.** Correlation between Positive Diametric Activity (PDA) and Functional Connectivity (FC) in experienced meditators (EM)

Region	BA	Voxels	MNI (*x*,*y*,*z* mm)	*p* value*
**Meditation state effects**				
EM Med PDA and EM FC				
Default Mode Network (DMN)^seeds 1 and 2^				
No correlation	N/A	N/A	N/A	N/A
Central Executive Network^seeds 3, 4, and 5^				
Inferior Frontal Gyrus	L 45	>200	–56,14,18	0.01
Superior temporal lobe	L 22	>200	–46,–16,8	0.03
Posterioir cingulate cortex	L 31	>200	–12,43,23	0.01
Inferior Parietal Lobule	L40	>200	–48,–64,50	0.02
**State-to-trait effects**				
Δ PDA and Δ FC				
Default Mode Network (DMN)^seeds 1 and 2^				
Posterior Cingulate Cortex	L 31	>200	0,–36,34	0.02
Precuneus	L 19	>200	–35,–74,34	0.02
Limbic lobe, uncus	L 20	>200	–28,–22,–34	0.03
Central Executive Network^seeds 3, 4, and 5^				
Medial frontal lobe	L 6	>200	–16,–12,60	0.02
Superior temporal lobe	R 22	>200	56,2,6	0.01

ROIs 1,2,3,4 and 5 from Fig. 1*Aa*. *nonparametric (1000 permutations) with height threshold *p* < 0.05 and cluster-size FDR-corrected *p* < 0.05.

**Table 4.** Correlation between meditation hours and Functional Connectivity (FC) in experienced meditators (EM)

Region	*R*	BA	Voxels	MNI (*x*,*y*,*z* mm)	*p* value*
**Meditation state effects**					
Default Mode Network (DMN)^seeds 1 and 2^					
Middle frontal gyrus	0.87	R 10	>200	45,44,12	0.02
Central Executive Network (CEN)^seeds 3, 4, and 5^					
No correlations	N/A	N/A	N/A	N/A	N/A
**State-to-trait effects**					
Default Mode Network (DMN)^seeds 1 and 2^					
No correlations	N/A	N/A	N/A	N/A	N/A
Central Executive Network (CEN)^seeds 3, 4, and 5^					
Posterior Cingulate Cortex	0.63	L 29	>200	–06,–44,14	0.03
**Trait effects**					
Default Mode Network (DMN)^seeds 1 and 2^					
No correlations	N/A	N/A	N/A	N/A	N/A
Central Executive Network (CEN)^seeds 3, 4, and 5^					
Medial Frontal Gyrus	0.87	L 10	>200	–06,44,8	0.01

ROIs 1,2,3,4 and 5 from Fig. 1*Aa*. *nonparametric (1000 permutations) with height threshold *p* < 0.05 and cluster-size FDR-corrected *p* < 0.05.

#### Correlations between hours of meditation practice and FC

##### DMN

**Figure 4.**
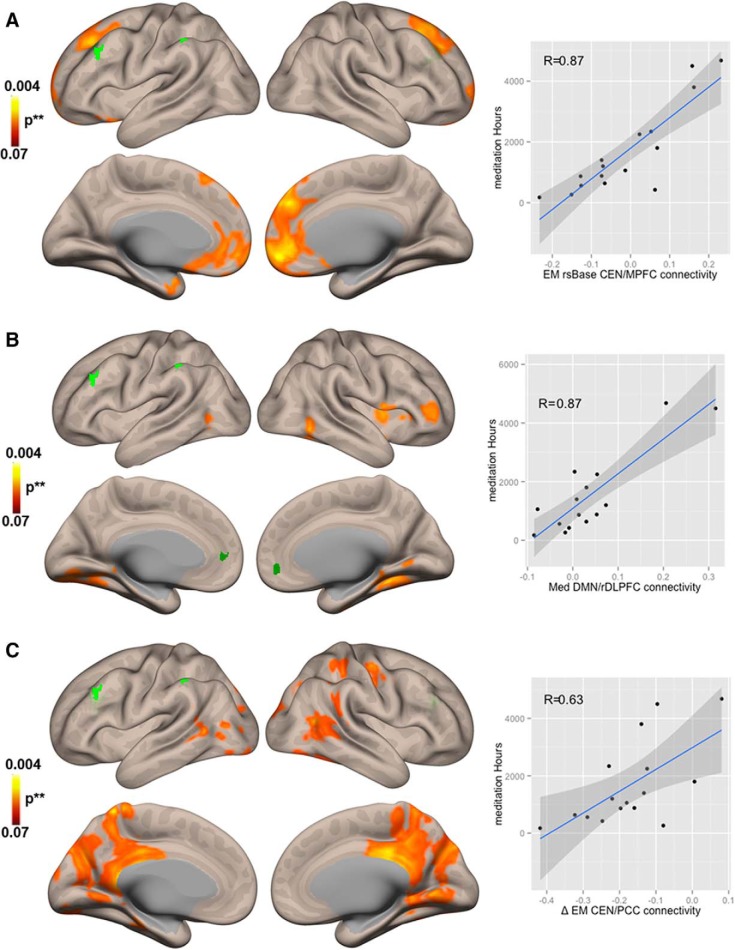
Correlations between meditation hours (MedHrs) and functional connectivity (FC). ***A***, Brain regions showing the correlation of MedHrs and FC at baseline for meditators. ***B***, Brain regions that show significant correlation between MedHrs and FC during the meditation state in meditators (EM Med). ***C***, Brain regions that show significant correlation between MedHrs and the change in FC during the transition from state-to-trait meditation in meditators (**Δ**EM = rsBase - rsPost). Dark green clusters at the Default Mode Network (DMN ROIs 1 and 2 from Fig. 1*Aa*) and bright green clusters at the Central Executive Network (CEN ROIs 3 and 4 from Fig. 1*Aa*) show in each case the seeds used to determine the estimated contrast. **nonparametric (1000 permutations) with height threshold *p* < 0.05 and cluster-size FDR-corrected *p* < 0.05.

Whole-brain FC analysis showed a significant correlation (*R* = 0.87) between the hours of meditation and the increase in connectivity between rDLPFC and MPFC during meditation (Fig. 4*B*).

##### CEN

No significant correlations were found.

#### Correlations between hours of meditation practice and PDA

No significant correlations were found for meditation trait, state, or state-to-trait PDA and meditation hours.

### Meditation state-to-trait effects

#### fALFF analysis

##### DMN

No significant differences were found.

##### CEN

The multilevel modeling one-way repeated measures ANOVA yielded that there was significant variation among rsBase, Med, and rsPost, χ^2^(2) = 24.57, *p* < 1e-04. The *post hoc* analysis showed that CEN fALFF was significantly increased in bilateral DLPFC (BA 9, Fig. 1*Ac*) during rsPost (mean = 0.09) compared to rsBase; mean = –0.55, b = 0.63, *p* = 0.006; red box plots (EM rsBase < EM rsPost) in Fig. 1*B*.

#### FC analysis

##### DMN

No differences were found.

##### CEN

EM showed increased connectivity (χ^2^(2) = 14.47, *p* < 0.001) with the Precuneus (BA 23) and right angular gyrus (BA 39) at rsPost compared to rsBase; Fig. 2*Ac* and red box plots (EM rsBase < EM rsPost) of Fig. 2*B*.

#### PDA analysis

##### PDA

The multilevel modeling one-way repeated measures ANOVA yielded no significant differences. However a two-sample paired *t* test showed a significant increase relative to rsBase; *t*_(15)_ = 1.97, *p* = 0.03, green box plots (EM rsBase < EM rsPost) of Fig. 1*B*, uncorrected.

#### Correlations between PDA and FC

##### DMN

There was a significant negative correlation (*R* = –0.70) between the change in PDA from rsBase to rsPost and the change in FC from rsBase to rsPost between MPFC (BA 10) and posterior cingulate cortex (PCC; BA 31), precuneus (BA 19), and limbic lobe (BA 20; Fig. 3*B*).

#### Correlations between hours of meditation practice and FC

##### DMN

No significant correlations were found.

##### CEN

Whole-brain FC analysis showed a significant correlation (*R* = 0.63) between the change in CEN and PCC connectivity from rsBase to rsPost and the hours of meditation practice (Fig. 4*C*).

#### Correlations between hours of meditation practice and PDA

No significant correlations were found for meditation trait, state, or state-to-trait PDA and meditation hours. For this and all other significant correlation analysis coordinates, see Table 4.

## Discussion

Meditation trait was characterized by a significant reduction in activity and FC within the DMN and increased anticorrelations between DMN and CEN in EMs. However, the latter anticorrelations were only present in meditators with less than three years of practice. Conversely, the meditation state and the meditation state-to-trait periods showed increased activity and FC within the DMN and between DMN and CEN. While there were no meditation trait PDA metric differences, we found significant increase during meditation state that persisted in meditation state-to-trait. The gradual reconfiguration in DMN and CEN suggest a neural mechanism by which the CEN negatively regulates the DMN and is probably responsible for the long-term trait changes seen in meditators and reported psychological well-being.

To our knowledge, no previous study has directly compared trait, state, and state-to trait conditions in EMs using fALFF and FC. In so doing, our findings demonstrate the following for each stage.

### Meditation trait

We found that meditation trait is characterized by (1) a significant reduction in brain activity of specific nodes of the DMN, most prominently the MPFC and MTG as well as CEN nodes in the right and left DLPFC and (2) a reduction in FC, both within DMN and between DMN and CEN. Thus, the former reduction in intrinsic DMN connectivity would speak for trait reduced synchrony of DMN regions for meditators and is in accordance with previous studies (Hasenkamp and Barsalou, 2012; Doll et al., 2015) which found that a region in MPFC showed decreased connectivity with the PCC in mindfulness experts compared to novices. Similarly, the latter finding of reduced FC between DMN and CEN for meditators in the baseline resting state is in accordance with two recent studies (Doll et al., 2015; Kemmer et al., 2015), suggesting a critical interplay between DMN and CEN for repeated engagement of attention on present moment experience. Hence, DMN activity and FC, instead of being engaged in task-unrelated thought or mind-wandering, resulting in activation and synchronization of the DMN (Mason et al., 2007), rather is again and again suppressed to re-engage in present moment awareness and hence reflects the stronger anti-correlated coupling between CEN and DMN (Mooneyham et al., 2017; Marusak et al., 2018). This is idea is in accordance with preliminary analysis using dynamic FC on the meditation state data that suggest that meditation trait is characterized by more transitions between brain states (mindful vs mind-wandering) over time, and thus meditators probably spent overall less time in a mind-wandering or a mindless state (Martínez et al., 2019). However, and most importantly, the additional finding of a positive correlation between meditation experience and increased connectivity between CEN and MPFC, which also has been reported in other studies comparing novice versus expert meditators (Brewer et al., 2011; Jang et al., 2011; Shaurya Prakash et al., 2013) and, which at first seems to contradict the increase in anticorrelations for meditators in general, rather is a subtle difference that points to additional network reconfiguration occurring as practice increases for expert meditators (Brewer et al., 2011; Hasenkamp and Barsalou, 2012). We then disentangled this finding by searching for the differences between intermediate (<1130 h of practice, approximately three years of 1 h daily practice) and more EMs (>1130 h) FC in these nodes. The finding, hence suggests that the trait state of intermediate meditators is characterized by a stronger reduction in DMN connectivity and significant increases in anticorrelations between CEN and MPFC. In more EMs (>1130 h of practice) the reduction in DMN connectivity still remains, however the anticorrelations have returned to a pre-meditation state. This finding, we think, is of major importance and points to the evolution of brain activity and connectivity changes as meditation progresses from an intermediate to more advanced stages, and that this change is rather a slow one, with the final sole reduction of DMN activity and connectivity independent of CEN suppression of at least three years of practice and can even evolve to a sustained increase in connectivity between CEN and DMN in meditators with more than three years of experience (Brewer et al., 2011; Creswell et al., 2016). Additionally, we think that this trait suppression of DMN in more EMs independent of anticorrelations with the CEN, is the ultimate network reconfiguration without any active and repeated suppression of the DMN by the CEN and thus, a more effective, consolidated effortless baseline, reflecting a completely transformed and stable mindful state with suppression of DMN without the more active and repeated transitions between mindful versus mind-wandering brain states in intermediate meditators. However, this hypothesis has to be tested in longitudinal studies.

### Meditation state

We found that meditation state is characterized by the following: (1) increase in the activity of specific CEN nodes (bilateral DLPFC), (2) increased FC between CEN nodes and DMN nodes (MPFC and PCC), (3) increased PDA, (4) PDA positively correlates with the strength in FC between the CEN and the DMN, and (5) that meditation experience (meditation hours) positively correlates with connectivity between MPFC and right DLPFC during meditation. These findings are in accordance with previous literature (Brewer et al., 2011; Hasenkamp and Barsalou, 2012) and suggest that the neural mechanisms underlying the active meditation state is associated with differential activation and connectivity of CEN nodes modulate activity and connectivity of the DMN. Importantly, although we cannot rule out that other brain networks mediate the effect of CEN on the DMN, our results indicate that activity in the CEN, specifically in the DLPFC effects on the activity and connectivity of the DMN hub nodes, i.e., MPFC and PCC. This hypothesis is in line with previous meditation studies (Brewer et al., 2011; Hasenkamp and Barsalou, 2012; Hasenkamp et al., 2012) but is of particularly interest given a study by Chen et al. (2013), where they used TMS and fMRI to demonstrate a directional causal relationship by which a DLPFC node situated within the CEN inhibits the MPFC portion of the DMN. This in turn is further supported by evidence of monosynaptic projections between CEN and DMN structures in rhesus monkeys, which indicate that these efferent pathways are part of an elaborate anatomic circuit which could mediate aspects of attention, memory, and external or internal perception (Selemon and Goldman-Rakic, 1988). What is more, recent evidence has shown that there is a direct relation between GABAergic inhibition within the mPFC and the reactivity of amygdala during emotional processing. Delli Pizzi et al. (2017) investigated the mPFC-amygdala circuit both with resting-state fMRI (rs-fMRI) and proton MR spectroscopy (MRS) as well as trait anxiety. Their results showed that the rs-fMRI signals of the amygdala and the mPFC were significantly anti-correlated and that this negative functional coupling between the two regions was inversely correlated with the GABA+/tCr level within the MPFC and the STAI-Y2 scores. This suggests a close relationship between MPFC GABA levels and functional interactions within the MPFC-amygdala circuit. Although this is still an open question and needs additional research with simultaneous MRS acquisition during and after meditation, we propose that a meditation state: (1) increases activity within the CEN, (2) this increase in CEN activity directly downregulates the two major DMN nodes (i.e., MPFC and PCC), (3) this down regulation of MPFC is coupled with a reduction in GABA+/tCr levels and reduced amygdala reactivity which, and (4) have a direct impact on anxiety scores and the physiology of emotion regulation.

### Meditation state-to-trait

We found that the meditation state-to-trait is characterized by shoeing remnants of the meditation state effect both in activity and connectivity. Specifically, (1) increased activity of the CEN; (2) increased FC between CEN nodes and DMN, although only with the PCC, which has been suggested to be more related to internal meditation practices (Scheibner et al., 2017); and (3) an increased PDA. Moreover, the change in PDA from rsBase to rsPost was negatively correlated with change in intrinsic connectivity of the DMN (MPFC and PCC), i.e., the greater the increase in PDA the less the intrinsic connectivity of the DMN at rsPost. This is also in line with previous studies that suggest that the post-task resting-state network activity and connectivity reflect an aspect of the immediately preceding brain state (Waites et al., 2005) and that these brain changes support the role of learning from a recently performed task as a concomitant process in expertise development (Muraskin et al., 2016). Hence, it further supports the hypothesis that the activity within CEN nodes (specifically DLPFC) actively suppresses DMN nodes and that this suppression carries over during a restful state after meditation, although it shifts to a more posterior node, namely the PCC, probably because this node is more engaged in this particular type of meditation, i.e., internal focused meditation (Scheibner et al., 2017). Finally, we found that meditation experience positively correlates with change in CEN-DMN connectivity. Hence, the more EMs seem to have a larger homeostatic rebound after meditation in CEN-DMN connectivity (for a discussion of this hypothesis, see below).

Finally, and because of the design of the present study, we propose that both, the increased PDA as well as the increased connectivity between DMN and CEN during meditation, as well as the remnants after meditation, at the beginning of practice, effectively produce a “homeostatic rebound” to what we identify as the meditation trait, namely the reduced activity and connectivity within DMN and between DMN and CEN. We think that brain activity, brain connectivity and long-term ensurance of homeostasis depend on intrinsic properties that determine the functionality of these neuronal networks. Homeostatic factors are inherently important and involve complex self-regulatory mechanisms (Davis, 2013). Consequently, it seems reasonable to view this homeostatic rebound as the plasticity that results from a repeated meditation practice within a context of otherwise stable network configurations. First, without the existence of potent mechanisms that perturb this normal balance between networks, and secondly, similarly potent mechanisms that in turn stabilize this perturbation, our capacity to learn and change brain function would be lost. Hence, we suggest, that there is a two stage reconfiguration or homeostatic plasticity (Davis, 2013; Hellyer et al., 2017), that evolves with meditation practice. First, as meditation practice begins, it produces stronger anti-correlated coupling between CEN and DMN (Mooneyham et al., 2017; Marusak et al., 2018) and thus increases the frequency of repeated periods of DMN suppression, even without being actively engaged in meditation, resulting in increased number of moments during the day of engagement in present moment awareness and less mindlessness. This initial change is in accordance with additional evidence showing that increased anticorrelations between DMN and CEN are associated with a healthy development (Chai et al., 2014) and aging (Keller et al., 2015; Esposito et al., 2018), cognitive reserve (Franzmeier et al., 2017), superior cognitive performance (Chai et al., 2014), reduced risk of psychopathology (Whitfield-Gabrieli et al., 2009; Andrews-Hanna et al., 2014), increased emotional stability (Servaas et al., 2017), and overall physiologic and psychological well-being (Fountain-Zaragoza and Prakash, 2017). As meditation increases, a second reconfiguration occurs where the frequency of repeated periods of DMN suppression start to overlap and rather become prolonged periods of engagement in present moment awareness up until the point where it is just continued present moment awareness. Once this point has been reached, we think that there is no more need of active monitoring if one is in a mindless state or mindful state and thus the anticorrelations start to return to a normal pre-meditation level.

The plasticity resulting from a repeated meditation practice, i.e., the suggested homeostatic rebound, may also be the underlying mechanism of many of the benefits reported with meditation practice (Goldberg et al., 2018) that depend on the top-down regulation of the DMN by CEN (Chen et al., 2013; Garrison et al., 2015). In doing so, it is possible that functional abnormalities in cortical and subcortical regions involved in emotion regulation such as anxiety, depression, or other types of psychopathology that relate to deficiencies in noradrenergic and serotonergic function are also recalibrated and thus enhancing brain noradrenergic or serotonergic transmission (Whitfield-Gabrieli and Ford, 2012; Willner et al., 2013). Meditation training, thus, with time, may lead to observable changes in the brain and in neurotransmitter levels (Guglietti et al., 2013; Jindal et al., 2013) accounting for its antidepressant effects and use in psychotherapy. However, this last hypothesis has to be corroborated with further studies that particularly look at this process.

We also want to note some limitations of this study. First off, when we talk about “activity” in the present study we do this with complete knowledge of the limitations of BOLD imaging as the standard technique used to generate images in fMRI studies, and that relies on regional differences in cerebral blood flow to indirectly delineate regional activity (Arthurs and Boniface, 2002; Huettel, 2004). Thus, activity in the present study refers to BOLD activity. There were a modest number of participants. In turn, this motivated a ROI approach focused on specific nodes from the DMN and CEN, based on the prior literature, so that a conservative level of statistics could be employed. A strength of this study is that it represents a first step in elucidating the potential neurobehavioral mechanisms mediating the practice of meditation on DMN activity and the FC. Specifically, it suggests that homeostatic rebound of anticorrelated DMN and CEN networks after meditation could be the compensatory mechanisms operating in the brain that may account for the trait changes in meditators.

## Conclusion

Here, we examined the brain changes underlying the state-to-trait experience and training of meditation. We focused on the activity and connectivity of the default and executive networks given their respective roles in internal cognition, self-regulation, and awareness. The findings presented reveal that active meditation practice deliberately engages networks related to cognitive and attentional control that effectively directs the focus of attention and curbs our usual mode of getting carried away by the endless stream of internal and external distractions. Furthermore, we showed that this active state is carried over to an immediate and passive restful state with similar network relationships than the active state. The trait effects of meditation suggest a recalibration and reconfiguration of network structure, or homeostatic plasticity (Davis, 2013; Hellyer et al., 2017), that produces in particular reductions in DMN activity and connectivity. However, the way this suppression is achieved depends on the stage of meditation. Intermediate meditators show stronger anti-correlated coupling between CEN and DMN (Mooneyham et al., 2017; Marusak et al., 2018), which suggests increased frequency between states of mindlessness and mindfulness and thus moments of DMN suppression, even without being actively engaged in meditation. Finally, with more experience in meditation, the moments of mindfulness become so frequent that no more transitions are needed and thus become the new default mode with sustained reduction in DMN activity and connectivity without effort. These, we think are the trait characteristics that ultimately underlie the beneficial effects of meditation, yet allowing the initial practitioner to increase the number of moments of mindful and clear reality to finally achieve a sustained mindful state, even to see reality as it actually is, without the perturbing vail of past or future illusion.

## Synthesis

Reviewing Editor: Bradley Postle, University of Wisconsin Decisions are customarily a result of the Reviewing Editor and the peer reviewers coming together and discussing their recommendations until a consensus is reached. When revisions are invited, a fact-based synthesis statement explaining their decision and outlining what is needed to prepare a revision will be listed below. The following reviewer(s) agreed to reveal their identity: Sungshin Kim.

Here are the full narrative reviews:

Reviewer #1:

The manuscript is written well enough for comprehension but there are different misspellings and grammatical errors, so a revision of an expert in English editing is recommended.

The results summarized in the abstract should be described more clearly (were the comparisons performed between groups or across conditions?)

Which was the rationale of using fMRI sequences with different parameters for resting state and meditation acquisition? Although it was stated that the data sets with the shorter TR were finally truncated at 151 volumes to match the resting state acquisition this should be better clarified in both methods and limitations discussion.

At line 199 the numerator of fALFF should be (0.01-0.1 Hz).

The sentence at lines 214-216 : “We first obtained the meditation trait differences of the fALFF maps of HC and EM at baseline (rsBase) using two-sample t-tests. The thresholded images were then converted into binarized masks in order to extract the mean fALFF maps for all subjects and states” appear to be a repetition.

Was the Fisher transformation applied to fALFF values prior to perform the t-tests? Also, the Fisher transformation is usually reported for correlation values, I'm not sure it can be applied to every value ranging from -1 to 1.

The sentence at line 261: “as opposed to global signal regression, a widely used preprocessing method known to artificially introduce negative correlations” is redundant as the concept has been already clarified in the same paragraph.  

Which is the rationale for using different bands for temporal filtering (in some sections it is 0.01-0.1 Hz, in others 0.008-0.09 Hz or 0.009- 0.08 Hz)?

The limitation of the BOLD technique in giving information about “activity level” should be pointed out in the discussion. Indeed, even the ALFF approach is a measure of signal fluctuation amplitude, not of a steady state activity.

Finally, there are recent studies investigating the role of medial prefrontal cortex with GABA/Glutamatergic neurotransmission in situations that meditation is supposed to relieve, such as anxiety, that can be taken into account when discussing the results.

Reviewer #2:

This manuscript presented a fMRI study showing a gradual configuration of central executive and default mode networks from pre-meditation, during meditation, and post-meditation. For this, the authors designed back-to-back fMRI experiments with experienced meditators, and a control experiment of pre-meditation resting state scanning with novice healthy participants. The authors characterized network configuration using two measurements, fALFF which is power of low-frequency components of BOLD and functional connectivity (FC), then these were related with meditation experiences. The manuscript contributes to the literature as it provides novel data sets regarding how networks are reconfigured by meditation.

Using a novel back-to-back fMRI experiment design, the authors elucidated neural mechanism of meditation, showing how the default mode network interacts with the central executive network. The correlation results with high goodness of fit are very impressive. Also, their new suggestion regarding homeostatic rebound of anticorrelated DMN-CEN networks after meditation is an interesting hypothesis worthy of further investigation. However, I have several concerns in the experiment design and analysis that should be addressed for publication.

Major points:

1. For experienced meditators, the authors compared three data sets, pre-meditation, during-meditation, and post-meditation. My major concern is discrepancy of scanning protocols between pre-/post-meditation and during-meditation in terms of TR, scanning length, and so on. The authors briefly mentioned they used the initial portion of data for during-meditation to match the length of pre-/post-meditation data, 151 volumes (in line 281). Because they used different TR, 2 sec vs. 1.5 sec for pre-/post-meditation and during-meditation, the actual duration in time was different given the same number of volumes. Especially, due to the different TRs, the frequency ranges were also different as they described in line 205 and 206. This affects the calculation of fALFF and may induce bias of lowering fALFF of during-meditation, acquired by higher TR (larger range of frequency), than that of pre-/post-meditation.

However, there was no explanation why they used different protocols. If this was done by mistakes, they should provide quantitative evidences that difference in protocols had no significant effects on the results. They also need to explain why only the initial portion of the data was used. What if using the entire data of duration-meditation? Will it still support their conclusions, at least regarding the results for during-meditation?

2. Another major concern is the definition of ROIs, which I think unclear (lines 209-216). As I understand, the ROIs were marked as green solid circles in Figure 2A and these were obtained from the 2-sample whole-brain t-test between fALFF maps of HC rsBase and EM rsBase. The whole-brain t-test results were shown in Figure 1A and the significant clusters were intersected with CEN and DMN masks adapted from a previous study.

However, this seems not the case in Table 1. I guess clusters marked with superscripts (1-6) are the ROIs which were used as seeds in subsequent FC analysis. However, ROIs in CEN were listed under Meditation State-To-Trait Effects and two ROIs (bilateral IPL) marked as 5 and 6 were not shown in the table, which I did not expect. The authors explicitly described the target ROI's were obtained from the two sample t-test between HC rsBase vs. EM rsBase. Additionally, it is unclear whether they used peak coordinates (maximum t-value) and generated spherical ROIs from the coordinate or used the entire clusters. All the results were dependent of the ROIs in DMN and CEN because the authors described their results based on these.

The author summarized the results separately for DMN and CEN. Were all the results of fALFF analysis and FC analysis calculated by taking the mean of two ROIs for each of DMN (figure 1.A.1 and 1.A.2) and CEN (figure 1.A.3 and 1.A.4)? Then, why did the author not use ROIs in CEN marked as 5 and 6, which correspond to bilateral IPL.

3. It is unclear how the authors calculated correlation between PDA and FC for different regions in the same network. For example, I do not understand why R^2 (maybe R is correct) is exactly the same for all the region in the same network in Table 3.

4. The authors report correlations between FC and hours of meditation as well as PDA. I wonder if there is any significant correlation between hours of meditation and PDA.

5. There is no explanation for the whole-brain analysis shown in figure 4.A. The authors described a correlation result only.

6. I suggest to reorganize the result section incorporating the correlation results as separate analysis under three effects. Specifically, it would be better to move sections 3.4 and 3.5 under 3.1-3.3 so that results of trait (3.4.1. and 3.5.1), state(3.4.2. and 3.5.2), and state-to-trait effects (3.4.3. and 3.5.3) can move to 3.1.4, 3.1.5, 3.2.4, 3.2.5, 3.3.4, 3.3.5.

By doing this, the result section will be more organized in the order of trait, state, and state-trait effects.

7. In discussion, interpretation of their results is rather difficult to understand. Specifically, I do not understand how more transition between mindful vs. mind-wandering is related with stronger anti-correlation. I wonder if the authors can show this more quantitatively using dynamic connectivity analysis with their data sets. I think 20-minute fMRI is long enough to perform this analysis although the authors used only initial portion of data, which are only 151 volumes.

8. Other studies comparing novice vs. expert meditators showed increased connectivity between DMN-CEN. However, the current result found no significant difference after rebounding from the anticorrelation. The authors did not explain this discrepancy. Did the expert meditators in other studies have more experiences than meditators in the current study? Then, can we say connectivity between DMN-CEN continue increasing beyond the level of novice after rebounding from anti-correlation?

Minor points:

1. Please give a reference for Free Cluster Enhancement (TFCE) method in line 213.

2. The authors introduced a new measurement, PDA based on the hypothesis of CEN negatively regulating the DMN (lines 221-226). However, it would be helpful to conceptualize PDA in a way of giving better insight into understanding their results.

3. Were the signals from CSF and the white matter regressed out when calculating FC as described in lines 266-268?

4. typo in line 289, “differents” -> different

5. typo in line 303, left Superior Frontal Gyrus (MFG) -> left Superior Frontal Gyrus (SFG)

6. In the description of ANOVA results (e.g., line 313), what do you mean by “Meditators”? I think this should be three experimental conditions, pre-, during, post-meditation. Meditator seems to mean subjects, which I think not.

7. I am not sure why there is no ANOVA result in FC analysis of Meditation

8. In 3.2.2. FC analysis, MFG and ACC were not shown in figure 2.A. Only PCC and IPL were shown.

9. In post-hoc Tukey test, please explain the meanings of “M” and “b” (e.g., line 316).

10. In line 345 of 3.3.3. PDA, was the t-test result (p = .03) corrected for multiple comparison?

11. Which of R^2 and R is correct? The main text used R^2 but the figure used R. I guessed the latter is correct.

12. In line 369, the authors need to briefly explain why they divided EM group into two groups. They used an expression such as “to further disentangle this finding”, but the authors need to briefly remind that connectivity between CEN-MPFC at rsBase decreased from novice to experienced meditators, which seem contradictory to the finding. In discussion, the author did provide this explanation, but here it is little bit awkward to use the expression, “further disentangle” without any explanation.

13. What is the criterion of selecting 1130 hours differentiating experienced and intermediate meditators?

14. In discussion, line 390 describing the anticorrelations should be right after line 388 for better logical flow.

15. In line 486, Fist -> First

16. In line 515, there is a typo “ts”

17. In figure 2.A, the index of a color bar (red to yellow) may be wrong. -0.5 should be 0.5

18. What do black dots indicate in figure 1.B and 2.B?
